# Cadmium Exposure: Mechanisms and Pathways of Toxicity and Implications for Human Health

**DOI:** 10.3390/toxics12060388

**Published:** 2024-05-26

**Authors:** Fei Qu, Weiwei Zheng

**Affiliations:** 1 Key Laboratory of the Public Health Safety, Ministry of Education, Department of Environmental Health, School of Public Health, Fudan University, Shanghai 200032, China; feitsmc@163.com; 2 Center for Water and Health, School of Public Health, Fudan University, Shanghai 200032, China

**Keywords:** cadmium, oxidative stress, signal transduction, epigenetic changes, ADME, cellular toxicity, human health

## Abstract

Cadmium (Cd), a prevalent environmental contaminant, exerts widespread toxic effects on human health through various biochemical and molecular mechanisms. This review encapsulates the primary pathways through which Cd inflicts damage, including oxidative stress induction, disruption of Ca^2+^ signaling, interference with cellular signaling pathways, and epigenetic modifications. By detailing the absorption, distribution, metabolism, and excretion (ADME) of Cd, alongside its interactions with cellular components such as mitochondria and DNA, this paper highlights the extensive damage caused by Cd^2+^ at the cellular and tissue levels. The role of Cd in inducing oxidative stress—a pivotal mechanism behind its toxicity—is discussed with emphasis on how it disrupts the balance between oxidants and antioxidants, leading to cellular damage and apoptosis. Additionally, the review covers Cd’s impact on signaling pathways like Mitogen-Activated Protein Kinase (MAPK), Nuclear Factor kappa-light-chain-enhancer of activated B cells (NF-κB), and Tumor Protein 53 (p53) pathways, illustrating how its interference with these pathways contributes to pathological conditions and carcinogenesis. The epigenetic effects of Cd, including DNA methylation and histone modifications, are also explored to explain its long-term impact on gene expression and disease manifestation. This comprehensive analysis not only elucidates the mechanisms of Cd toxicity but also underscores the critical need for enhanced strategies to mitigate its public health implications.

## 1. Introduction

Cadmium (Cd) is a naturally occurring heavy metal that has become a global issue due to its extensive use in industrial production, especially in metal smelting, battery manufacturing, and as additives in plastics and pigments [1]. Although Cd naturally occurs in low concentrations, human activities have significantly heightened its levels in the environment, particularly in industrial zones and areas heavily affected by Cd emissions. Its persistence in the environment and its accumulative nature in biological organisms leads to widespread ecological and health issues. The primary routes of human exposure to Cd are inhalation and dietary intake, especially through Cd-contaminated water and food, because it can persist for long periods in water bodies and soils, transferring through the food chains [2]. Chronic exposure to low doses of Cd can cause a range of health issues, including kidney damage, osteoporosis, and effects on blood pressure and cardiovascular functions [3]. Different countries and regions have implemented a variety of regulatory measures to control the environmental release of Cd and reduce the risks of human exposure. However, challenges remain in significantly reducing the use and emissions of Cd due to its irreplaceable role in certain industrial processes.

Cd can disrupt cellular functions and cause tissue damage through multiple toxicological pathways, leading to various diseases and imposing significant economic burdens on society. Drawing on this understanding, this paper compiles and reviews a wide range of studies from the literature to comprehensively explore Cd’s toxic pathways and mechanisms. The aim is to provide a holistic perspective on the environmental and health risks associated with Cd, informing future research and guiding policy development.

## 2. The Absorption, Distribution, Metabolism, and Excretion (ADME) Process of Cadmium in the Body

Cd primarily enters the human body through respiratory and digestive pathways, especially in industrial settings such as metal smelting and battery manufacturing plants. It is mainly inhaled in the form of particulates or fumes, with the lungs having a relatively high absorption rate, ranging from 30% to 50% [4]. For the general population not exposed occupationally, food is the main source of Cd intake, accounting for over 90%, especially from crops grown in contaminated soil, shellfish, and other seafood [5]. In non-traditional situations, worn-out car tires release microplastic particles containing heavy metals, such as cadmium, into the environment. These particles can accumulate in soil and water, potentially entering the food chain and posing significant health risks to humans [6]. Smoking in enclosed spaces, such as cars or homes, further exacerbates the problem by significantly increasing the concentration of heavy metals in the air [7]. This results in higher levels of exposure for inhabitants through both direct inhalation and secondhand smoke. The situation is particularly concerning in smoking households, where the confined space and continuous exposure lead to a cumulative buildup of heavy metals in the body. This amplification of health risks underscores the importance of addressing both non-traditional sources and lifestyle factors contributing to cadmium exposure. The absorption rate of Cd through the gastrointestinal tract is comparatively low, generally between 3% and 7% [8], but can vary based on individual factors such as nutritional status. Adequate levels of essential minerals like iron, calcium, and zinc reduce cadmium uptake by competing for binding sites on transport proteins, while deficiencies increase absorption. Dietary components such as phytates and high-fiber foods bind Cd^2+^ in the gastrointestinal tract, decreasing absorption, whereas high-protein diets may enhance it. The integrity of the gastrointestinal lining, affected by nutritional status, also influences Cd absorption, with malnutrition increasing permeability and uptake. Additionally, nutritional status affects the expression of metal-binding proteins like metallothionein, which sequester Cd^2+^ and reduce its bioavailability.

Upon absorption into the human body, Cd is transported through the bloodstream and gradually accumulates primarily in the kidneys, liver, and bones. The kidneys are the main target organs for toxicity, with significant accumulation occurring in the renal cortex, particularly within the proximal tubule cells [9]. This accumulation predominantly results from the high reabsorption efficiency of Cd-complexes in the renal tubules, where Cd is filtered by the glomeruli and subsequently reabsorbed and accumulated. Long-term exposure can lead to kidney damage and, in severe cases, kidney failure. Additionally, it can interfere with Ca^2+^ metabolism, accumulated in the bones, and competitively replace bone minerals, leading to osteoporosis and other skeletal disorders over time [10].

Cd is not significantly metabolized within the body but predominantly remains in its unaltered form. Due to the high reabsorption efficiency in the renal tubules, cadmium primarily accumulates in the kidneys, especially in the renal cortex, for extended periods, leading to long-term effects. It can form stable complexes with proteins, such as metallothioneins (MTs), which reduce its reactivity and can be stored in the body for extended periods [11]. It is predominantly expelled from the body through the kidneys in urine, with prolonged exposure potentially impairing renal function and reducing excretory efficiency. With a biological half-life of 10 to 30 years, Cd can accumulate in the body for decades after absorption [1,12]. This prolonged accumulation means that even low-dose exposure also can ultimately lead to severe health damage.

Given the characteristics of its distribution and metabolism in the body, Cd levels in blood and urine are commonly used as biomarkers of exposure [13]. Urinary cadmium (U-Cd) primarily reflects the overall body burden of Cd and indicates long-term exposure levels, whereas blood cadmium (B-Cd) is more indicative of recent exposure [14]. Biomarkers of kidney damage, such as urinary *β*_2_-microglobulin (*β*_2_M), are employed to assess the impact of Cd exposure on renal function [15]. An increase in the activity of N-acetyl-*β*-D-glucosaminidase (NAG) in urine is a sensitive indicator of early kidney damage [16,17]. MTs, often measured in urine and blood, indicate the body’s adaptive response to Cd exposure as its levels rise. The decrease in δ-aminolevulinic acid dehydratase (δ-ALA-D) activity in urine, a typical marker for lead exposure, can also be influenced by Cd, especially during mixed heavy-metal exposure, making it a valuable early biomarker of cadmium’s biological effects [18].

## 3. Oxidative Stress Caused by Cadmium

Oxidative stress induced by Cd is a critical aspect of its toxicity mechanisms. It occurs when an increase in oxidants and a decrease in antioxidants leads to the accumulation of reactive oxygen species (ROS) and reactive nitrogen species (RNS). This imbalance can damage cellular macromolecules, such as lipids, proteins, and nucleic acids, extensively harming cell structure and function [19]. This series of steady-state imbalances can lead to disruptions in cellular signaling pathways and may also trigger cell apoptosis or necrosis. Furthermore, it can potentiate its oxidative stress effects by perturbing the cellular antioxidant defense system [20,21]. Key antioxidant enzymes like superoxide dismutase (SOD), glutathione peroxidase (GPx), and catalase (CAT) may experience diminished activities or inhibited expressions in response to Cd, thereby compromising the capacity to counter oxidative damage and intensifying the adverse consequences of oxidative stress in cells.

Upon entry into the cell, Cd^2+^ initially affects the mitochondria, which serve as the primary sites for cellular energy production and the major sources of ROS generation. Cd^2+^ interferes with the electron transport chain (ETC) by binding to thiol groups in Complex I (NADH dehydrogenase) and Complex III (coenzyme Q) [22]. This interaction disrupts the normal functioning of these complexes, reducing efficient electron transfer and increasing the risk of electron leakage. These leaked electrons can react directly with oxygen to form superoxide anions (O^2−^), highly reactive ROS that can further transform into other reactive species such as hydrogen peroxide (H_2_O_2_) and hydroxyl radicals (·OH), intensifying ROS production due to mitochondrial respiratory chain dysfunction caused by Cd [23,24].

Cd can induce a decrease in mitochondrial membrane potential, primarily resulting from disruptions in the ETC, which reduce the efficiency of proton pumps (Complexes I, III, IV, etc.) and consequently fail to maintain the essential proton gradient across the mitochondrial membrane [25]. The subsequent decrease in membrane potential not only hampers mitochondrial adenosine triphosphate (ATP) synthesis but also initiates mitochondrial-mediated apoptotic pathways, characterized by heightened permeability of the mitochondrial outer membrane [26,27]. This alteration in permeability facilitates the release of Cytochrome c (Cyt c) into the cytoplasm, thereby instigating a cascade of caspase activations [28].

Activation of Nicotinamide adenine dinucleotide phosphate (NADPH) oxidase (NOX) by Cd is another crucial mechanism for inducing oxidative stress and cellular toxicity. NOX, an enzyme complex found in various cell types, primarily generates ROS by transferring electrons from NADPH to oxygen molecules, playing a crucial role in immune cells like macrophages and neutrophils where the ROS are essential for killing pathogens [29]. However, in non-immune cells, such as renal cells, excessive activation of NOX can lead to oxidative stress, subsequently damaging cell structure and function. Cd directly interacts with NOX subunits, causing conformational changes that increase enzyme activity, possibly through interactions with specific amino acid residues like cysteine, which are critical for maintaining enzyme function [30]. Cd also influences the activity of transcription factors, such as Nuclear Factor kappa B (NF-κB) and Activator Protein 1 (AP-1), increasing the gene expression of NOX. These transcription factors are active in inflammatory responses, and their activation can lead to elevated mRNA levels of NOX subunits, thereby enhancing the synthesis and assembly of the enzyme complex. Additionally, Cd can indirectly enhance NOX activity by modulating intracellular signaling pathways like Protein Kinase C (PKC) and Mitogen-Activated Protein Kinase (MAPK) pathways, which are involved in cellular stress and inflammatory responses, thereby promoting increased ROS generation. Prolonged or high-dose exposure to Cd can damage cell membrane lipids through superoxide anions and generates toxic hydroxyl radicals via the Fenton reaction, destroying proteins and DNA and triggering apoptosis or necrosis [31]. Furthermore, oxidative stress can activate a series of downstream inflammatory responses, exacerbating tissue damage and dysfunction [32].

The extensive impact of Cd-induced oxidative stress on cellular structure and function is primarily mediated through three interconnected pathways: lipid peroxidation, protein oxidation, and DNA damage, all of which collectively contribute to cell damage and disease progression.

Lipid peroxidation, a significant hallmark of Cd-induced oxidative stress, involves ROS such as O^2−^ and H_2_O_2_, initiating peroxidation reactions in unsaturated fatty acids within cell membranes, compromising their structural integrity and leading to cellular dysfunction [33]. This process also generates highly reactive and toxic end products like malondialdehyde (MDA) and 4-hydroxynonenal (4-HNE), which can react with cellular macromolecules including proteins, lipids, and nucleic acids to form stable adducts, further impacting cellular functions [34]. For example, MDA can react with cellular protein components in cells, such as lysine, histidine, and cysteine residues, disrupting normal protein function and triggering cellular stress responses.

Protein oxidation is another key mechanism by which Cd-induced ROS impact cellular function. ROS can directly oxidize amino acid residues in proteins, particularly attacking sulfur-containing amino acids like cysteine and methionine [35]. This oxidation progression leads to protein misfolding, loss of enzymatic activity, disrupted signal transduction, and cytoskeletal damage. These changes severely impact the overall stability and functionality of cells, particularly in the nervous system where neurotransmitter release and signaling processes heavily rely on the structural and functional integrity of proteins [36].

DNA damage is one of the most severe consequences of Cd-induced oxidative stress, where the generated ROS not only cause base oxidation but also lead to DNA strand breaks and cross-linking. If such damage is not promptly and effectively repaired, it can result in gene mutations, chromosomal aberrations, and even cellular transformation into cancer [37]. Chronic exposure is associated with an increased risk of various cancers, partly due to the sustained DNA damage caused by increased intracellular ROS production. Additionally, DNA damage triggers various cellular repair mechanisms, including the p53 signaling pathway, which can lead to cell-cycle arrest or apoptosis [38].

Cd exacerbates oxidative stress by damaging cellular antioxidant defense mechanisms, severely impacting long-term cell survival and function. Key components of the cellular defense system, such as SOD, GPx, and CAT, are essential for protecting cells from oxidative damage by decomposing ROS, including O^2−^ and H_2_O_2_ [39]. However, Cd^2+^ suppresses the expression and activity of these enzymes, diminishing the cell’s ability to eliminate ROS. For example, research on liver cells revealed that Cd treatment significantly decreased the activities of SOD and CAT and increased the levels of lipid peroxidation products, demonstrating how Cd aggravates oxidative stress by impairing the antioxidant enzyme system [40]. Additionally, Cd inhibits the synthesis and promotes the depletion of glutathione (GSH), a crucial intracellular antioxidant molecule, further reducing intracellular GSH levels and weakening the antioxidant capacity of cells [41].

## 4. Cadmium Impact on Signal Transduction Pathways

Cd can disrupt cellular signaling pathways, resulting in complex and varied biological effects that significantly influence cell growth, differentiation, apoptosis, and stress responses. It interferes with crucial signaling pathways, including but not limited to the MAPK, NF-κB, and p53 pathways, either through direct or indirect mechanisms. The disruption impairs cellular functions and triggers specific biological responses across different cell types, diminishing the ability to adapt to environmental changes, aggravating pathological conditions, and ultimately impacting the overall health of the organism.

**Figure 1:**
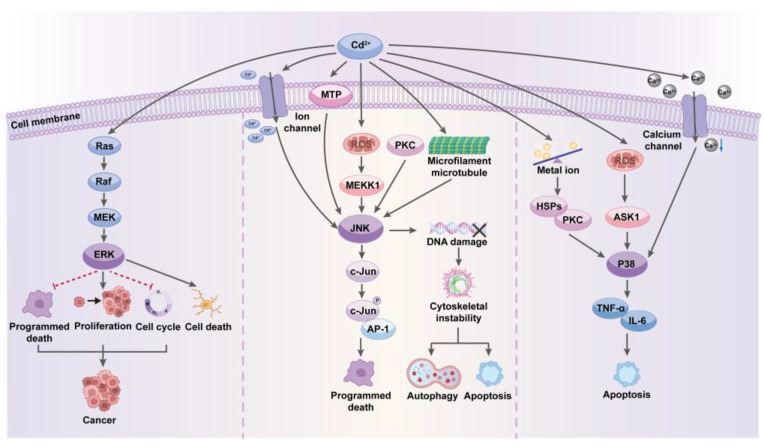
Cadmium’s Impact on MAPK Pathways: Disrupting Cellular Signaling and Health. The figure illustrates Cd^2+^ interference in the MAPK signaling pathways, highlighting direct and indirect effects on cellular function, resulting in abnormal cell responses and increased disease risk.

MAPKs are crucial for cells to respond to a variety of external signals, including growth factors, cellular stress, and inflammatory stimuli [42]. As shown in the Figure 1, Cd^2+^ can disrupt the pathway through several mechanisms. These include direct interactions with enzymes associated with the pathway and indirect effects on intracellular ROS levels [43]. Additionally, Cd may compromise the integrity of the cell membrane, altering membrane protein configuration and function, which in turn can activate or inhibit different branches of the MAPK pathway, affecting membrane receptor activity and downstream signal transmission, leading to abnormal activation or inhibition of the pathway [44]. Specifically, the MAPK pathway consists of three main branches: extracellular signal-regulated kinase (ERK), c-Jun N-terminal kinase (JNK), and p38 pathways, each of which responds specifically to different types of cellular stimuli.

The ERK pathway typically responds to growth factors and mitogenic stimuli. Under normal conditions, external signals initiate the Ras-Raf-MEK-ERK signaling cascade, activating ERK and promoting specific gene expression essential for normal cell growth and division, thereby preserving the physiological state and functionality of cells [45]. However, when Cd^2+^ persistently affects this pathway, it can lead to abnormal or sustained activation of the ERK pathway, resulting in aberrant signal transduction with varied biological outcomes across different cell types. In some cell types, sustained ERK activation can promote the development and progression of cancer, primarily due to continuous activation, which enhances cell proliferation, inhibits programmed cell-death mechanisms, and disrupts cell-cycle regulation [46]. The results lead to uncontrolled cell proliferation, supporting the growth and survival of tumor cells and thereby increasing the risk of cancer development. Moreover, Cd^2+^-induced excessive activation can lead to outcomes such as enhanced cellular stress responses, accelerated cell aging, or direct induction of cell death, particularly in neural and renal cells, effects that are closely linked to neurotoxicity and nephrotoxicity [25,47].

The JNK pathway is a crucial signaling pathway that significantly influences cellular reactions to diverse stress signals such as ultraviolet radiation, inflammatory factors, heat shock, and cytotoxins [48]. Cd^2+^ activates the pathway through multiple mechanisms, which induces a range of cellular responses, including the regulation of genes critical for cell survival, apoptosis, proliferation, and cell-cycle control by transcription factors. The activated pathway promotes the phosphorylation of the transcription factor c-Jun, a key step in cellular stress responses, aiding cells in adapting to or resisting the toxic effects of Cd [49].

Cd^2+^ can interact with specific receptors on the cell surface, such as MTPs and ion channels, to directly initiate signaling cascades that lead to JNK pathway activation, particularly evident in neural cells [50]. Additionally, by increasing the ROS level that acts to signal molecules, Cd^2+^ can activate upstream kinases in the MAPK pathway, such as Mitogen-Activated Protein Kinase Kinase Kinase 1 (MEKK1), further leading to JNK activation. Besides directly activating signaling pathways, Cd can also influence the activity of other crucial signaling molecules such as intracellular small GTPases and PKC, which are essential for regulating cellular stress responses and cell survival [51]. Consequently, it indirectly promotes JNK pathway activation through the modulation of these molecules. Cd may also affect the synthesis and degradation of specific proteins within the cell, particularly those involved in cellular stress responses and repair, by activating components of the JNK pathway such as AP-1, a transcription factor complex composed of c-Jun and other proteins, thereby influencing the cell survival state [52].

Furthermore, Cd can activate the JNK pathway by altering the dynamics of the cytoskeleton, which is crucial for cells to respond to mechanical stress and morphological changes, as it modifies the polymerization state of cytoskeletal proteins like microtubules and microfilaments, potentially leading to indirect JNK activation [53]. However, prolonged or high-dose exposure to Cd can lead to excessive activation of the JNK pathway, impairing cellular functions associated with various structural and functional disruptions, including DNA damage, cytoskeletal instability, autophagy, and apoptosis, which may ultimately contribute to tissue dysfunction, accumulate pathogenic alterations, and promote the development of diseases such as cancer, kidney disease, and bone disorders [54,55].

The p38 MAPK pathway, a pivotal signaling molecule, is activated by Cd^2+^ in response to immediate cellular stress, playing a crucial role in cell response to environmental stress, differentiation, and cycle regulation, ultimately influencing long-term survival through the modulation of inflammatory responses, apoptosis, and survival signals [56]. Similar to the activation of the JNK pathway, Cd^2+^ directly interacts with specific cell surface receptors or channels like calcium channels, triggering downstream signaling that alters intracellular Cd^2+^ concentrations, indirectly activating various calcium-related kinases and subsequently the p38 MAPK pathway.

An increase in ROS induced by Cd activates the p38 MAPK pathway either directly or indirectly through upstream kinases like apoptosis signal-regulating kinase 1 (ASK1), facilitating an oxidative stress response that helps cells resist the toxic effects of Cd [57]. Cd^2+^ can cause various changes in the cellular biochemical environment, such as alterations in pH and disruptions in cellular metal ion balance, influencing p38 MAPK activation through multiple pathways and affecting the expression and activity of specific stress proteins, like heat shock proteins (HSPs) and PKC, which can directly or indirectly activate p38 MAPK [58,59].

Furthermore, Cd can influence the production and release of cytokines through the activation of p38 MAPK, such as Tumor Necrosis Factor-alpha (TNF-α) and Interleukin-6 (IL-6), which are inflammatory factors playing a significant role in how cells cope with toxicity [60]. Additionally, p38 MAPK is linked to apoptotic pathways, and its activation can either promote or inhibit apoptosis, with the specific effect largely depending on the cell type and stress environment.

The NF-κB pathway is a central signaling pathway that regulates cellular responses to inflammation and immune reactions. Normally, NF-κB exists in an inactive state in the cytoplasm, bound to the inhibitory protein IκB [61]. Cd can activate the NF-κB pathway, mediating cellular responses to its toxicity by initiating inflammatory responses and modulating cell survival signals, but long-term or high-dose exposure may disrupt these protective mechanisms, thereby promoting the development of inflammation-related diseases and other chronic health issues.

As previously mentioned, one of the initial and most direct effects of Cd entering the cell is the increased production of ROS, which not only directly damages macromolecules like proteins, lipids, and DNA but also serves as a signaling molecule that participates in various intracellular signaling pathways, particularly activating the IκB kinase (IKK) in the NF-κB pathway. The result leads to the phosphorylation and subsequent degradation of inhibitor of κBα (IκBα), releasing the NF-κB transcription factor, allowing it to move into the nucleus and activate the expression of target genes, including various inflammatory factors and cell stress response genes [62]. Some studies have shown that Cd enhances NF-κB activity through a ROS-dependent pathway, intensifying inflammatory responses [25].

Cd can also mimic or activate cell surface receptors, such as tumor necrosis factor receptors (TNFR) and Toll-like receptors (TLRs), thereby activating associated adaptor proteins and signaling molecules like tumor necrosis factor receptor-associated factor 6 (TRAF6) and receptor-interacting serine/threonine-protein kinase 1 (RIP1), which in turn enhances the activity of the IKK, thereby activating the NF-κB pathway [63]. This mechanism allows cells to rapidly respond to external toxic stimuli, attempting to counteract their harmful effects through inflammatory responses.

Moreover, Cd directly activates or enhances the activity of various signaling molecules, including PKC, MAP kinases, and Phosphoinositide 3-kinase (PI3K), then promoting the activation of IKK or other regulatory mechanisms associated with NF-κB activity, directly or indirectly facilitating the activation of the NF-κB pathway [62]. For example, the activation of PKC can directly promote NF-κB activation, enhancing cellular survival signals in response to toxicity.

**Figure 2:**
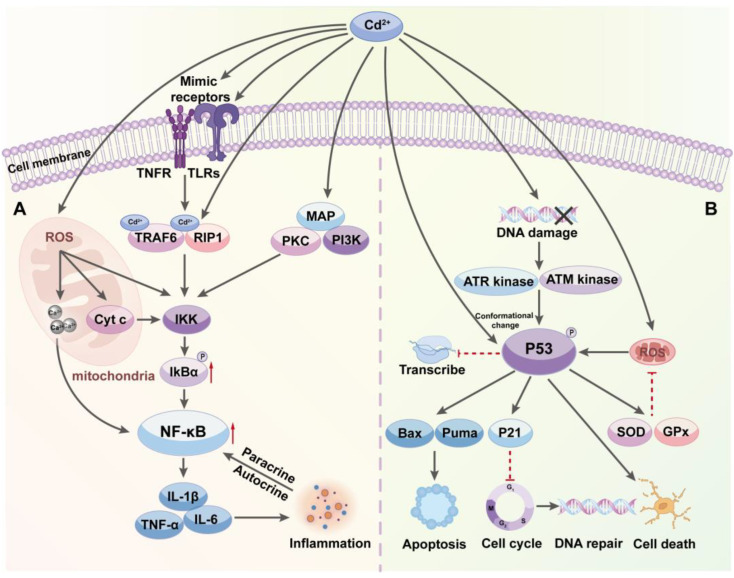
(**A**) Cadmium’s Role in Stimulating NF-κB Signaling: A Pathway to Enhanced Inflammation. This chart details Cd^2+^ activation of the NF-κB pathway, highlighting interactions with ROS, receptors, and kinases that lead to increased inflammation and potential chronic conditions. (**B**) Cadmium’s Impact on p53 Activation: Pathways to Cellular Stress Response and DNA Integrity. The figure illustrates Cd^2+^ effects on the p53 pathway, showing interactions that lead to DNA damage responses, including apoptosis and cell-cycle control, emphasizing the role of p53 in safeguarding cellular health.

Cd can also indirectly affect the NF-κB pathway by disrupting mitochondrial function, leading to excessive production of ROS and release of other stress mediators within the mitochondria, such as Cyt c, which can activate cell-death pathways and the IKK, thereby further promoting NF-κB activation [64]. Additionally, mitochondrial damage may affect intracellular Ca^2+^ levels, indirectly affecting NF-κB activity. Cytokines activated through the NF-κB pathway, such as TNF-α, IL-1β, and IL-6, can further amplify inflammatory signals through paracrine or autocrine mechanisms, thereby enhancing NF-κB pathway activation [65], ultimately leading to sustained inflammation and tissue damage due to the expanded toxic effects in the cells and tissues (Figure 2A).

The p53 protein, often hailed as the guardian of the genome, is a crucial tumor suppressor that plays a vital role in helping cells respond to DNA damage and other stress conditions by maintaining cellular genetic stability and preventing cancerous transformations through multiple downstream effector pathways, including cell-cycle arrest, apoptosis, DNA repair, and cellular senescence [66]. Cd^2+^ can influence the activity of the p53 pathway in several ways, including directly interfering with the stability of p53 protein and indirectly enhancing its transcriptional activity through the promotion of DNA damage.

Inside cells, Cd directly interacts with the p53 protein, altering its molecular conformation and function, which includes inhibiting its DNA-binding activity and suppressing its transcriptional regulatory capabilities [67]. Additionally, it can increase cellular oxidative stress responses, thereby indirectly activating the p53 pathway. Once activated, p53 induces the expression of various DNA repair enzymes and proteins that promote apoptosis and cell-cycle arrest. If the damage cannot be repaired, p53 could guide cells towards programmed cell death to prevent the malignant transformation of damaged cells [68].

Cd^2+^ closely interacts with DNA molecules, causing strand breaks and base damage primarily through chemical interference with the phosphate backbone and base pairing, which hinders normal DNA replication and transcription processes, leading to distortion of genetic information. Cellular repair mechanisms can recognize the DNA damage, activating complex DNA damage response (DDR) mechanisms, which trigger phosphorylation and activation of p53 [69]. Kasten et al. discovered that Cd activates the ATM and ATR kinases, which subsequently activate p53, leading to its rapid phosphorylation upon detecting DNA damage, thus facilitating a prompt cellular response to genotoxic stress that includes inducing cell-cycle inhibitors like p21 and effectively halting the cell cycle at the G1/S and G2/M checkpoints to permit necessary DNA repair [70]. Additionally, p53 activates multiple apoptotic pathways by upregulating pro-apoptotic factors such as Bax and Puma, steering cells towards programmed death to prevent potential malignant transformation of damaged cells [71]. This mechanism effectively mitigates tumor risks caused by genetic damage, showcasing the core role of the p53 in maintaining cellular and tissue stability.

In this way, p53 ensures that only cells with intact genetic information can continue to grow and reproduce, thereby maintaining organism health and genetic stability [72]. Under a Cd^2+^ exposure scenario, excessive production of ROS not only directly damages DNA but also stresses the cellular antioxidant system, thereby activating p53, which combats this toxic effect by enhancing the expression of intracellular antioxidants such as SOD and GPx, boosting the capacity to clear ROS. Thus, Cd activation of the p53 pathway by increasing ROS generation is not only a defense mechanism against cellular damage but also a critical process in regulating cell fate (Figure 2B).

**Figure 3:**
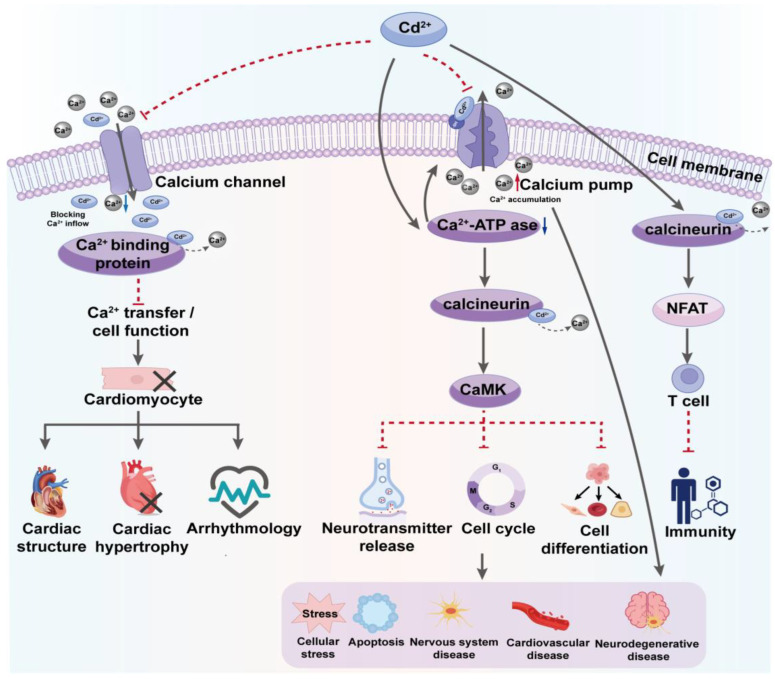
Disrupting Ca^2+^ Homeostasis: Cadmium’s Impact on Cellular Calcium Mechanisms. The diagram shows how Cd^2+^ disrupts Ca^2+^ homeostasis by mimicking and interfering with Ca^2+^ in cells. It details the effects on calcium channels and pumps, elucidating the subsequent risks to cell function and activity.

Ca^2+^ serves as a ubiquitous intracellular second messenger that regulates numerous biological processes, including neurotransmitter release, muscle contraction, cell growth, differentiation, and programmed cell death [73]. Under normal physiological conditions, cells use a precise signaling regulation network to maintain the concentration and distribution of Ca^2+^, ensuring the proper functioning of biological activities. However, the presence of Cd^2+^ disrupts this regulatory mechanism, disturbing the homeostatic balance of Ca^2+^ and triggering a range of physiological and pathological changes (Figure 3).

Cd^2+^ can directly interact with calcium ion channels on the cell membrane, such as L-type calcium channels, which play crucial roles in the heart and smooth muscle cells by regulating excitation–contraction coupling. Cd^2+^ can mimic Ca^2+^ to enter cells through calcium channels, impeding the normal influx of Ca^2+^ and competing with it for binding to intracellular calcium-binding proteins once inside, thus disrupting normal calcium signaling and calcium-dependent cellular functions [74]. This disruption is particularly detrimental to the electrophysiological stability of heart cells and the normal functioning of smooth muscle, altering myocardial contractility and smooth muscle tension, thus increasing the risk of related diseases. Moreover, chronic exposure may lead to lasting changes in heart structure and function, such as cardiac hypertrophy and arrhythmias [75].

Another significant mechanism through which Cd^2+^ disrupts Ca^2+^ balance involves its effect on cellular calcium pumps, impairing their function and altering the intracellular distribution of ions. Calcium pumps, especially Ca^2+^-ATPases, are primary carriers that regulate Ca^2+^ concentration by transporting it from the cytosol back out of the cell or into organelles like the endoplasmic reticulum and mitochondria, maintaining a dynamic equilibrium of calcium ion concentration across the cell [76]. Cd^2+^ can inhibit these pumps’ activity by directly interfering with this critical physiological process. This toxic effect is primarily mediated through interactions with key structural components of the pumps, especially thiol groups, which play essential roles in maintaining the structural and functional integrity of their active sites. By altering their conformation and function, Cd^2+^ could impair their ability to bind and pump Ca^2+^, resulting in an abnormally high intracellular concentration.

The inhibition of Ca^2+^-ATPase activity by Cd results in elevated intracellular Ca^2+^ levels, deteriorating various calcium-dependent physiological functions and disrupting cellular pathways that rely on them, specifically affecting key regulatory proteins such as calcium/calmodulin-dependent kinases (CaMK) and calcineurin [77]. To be more specific, CaMK, a series of functionally important kinases, is involved in regulating neurotransmitter release, cell-cycle control, cell differentiation, and various other cellular functions. Cd inhibits these crucial physiological processes by interfering with the activation and functionality of CaMK [78,79]. Calcineurin, primarily involved in regulating transcription factors such as NFAT during immune responses and cellular reactions, can be inhibited by Cd, a disruption that may impair T-cell activation and other immune functions, potentially leading to immunosuppression or the onset of other immune-related diseases [77].

## 5. Cadmium-Induced Epigenetic Alterations

Cd exposure is closely associated with epigenetic changes that regulate gene expression without altering the DNA sequence, profoundly affecting cellular functions and physiological processes. It can alter the activity and expression of DNA methyltransferases, resulting in changes in global or gene-specific methylation levels that are often linked to gene silencing, particularly in genes involved in cell-cycle regulation, apoptosis, and DNA repair [80]. Studies have shown that long-term exposure to Cd can increase the methylation of certain tumor suppressor genes, thus suppressing their expression and potentially promoting cancer development [81,82].

Cd can influence chromatin structure and gene expression by modifying histone post-translational modifications, such as acetylation, methylation, and phosphorylation, collectively dictating chromatin accessibility and thus modulating gene transcription activity [83]. For example, Cd has been found to increase methylation levels at specific histone sites, resulting in tighter chromatin structure and suppressed gene expression [84]. Additionally, it can reduce the expression or activity of histone acetyltransferases, decreasing histone acetylation and further affecting gene activity [80].

**Figure 4:**
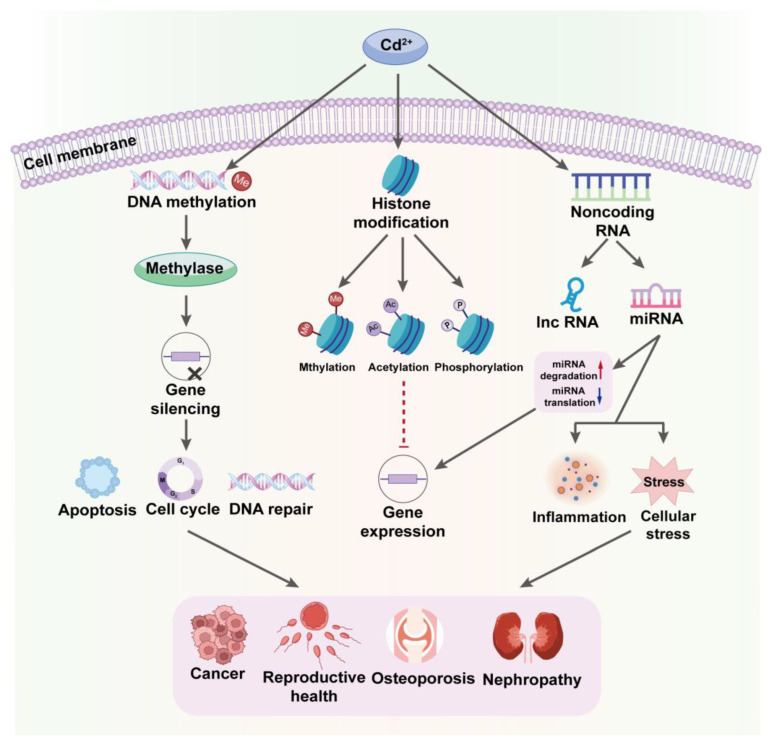
Cadmium-Induced Epigenetic Alterations: Impacts on Gene Regulation and Disease. This chart visualizes the pathways through which Cd^2+^ influences epigenetic processes, impacting DNA methylation, histone modification, and non-coding RNA activity. These alterations lead to significant changes in gene expression, affecting cellular functions and contributing to disease progression, particularly in cancer and other serious health conditions.

Cd modulates the expression and function of non-coding RNAs, such as long non-coding RNAs (lncRNAs) and microRNAs (miRNAs), which govern the expression of target genes. Typically, miRNAs regulate gene expression by binding to the 3′ untranslated regions of target mRNAs, thereby either inhibiting their translation or promoting their degradation. Cd can alter the expression patterns of specific miRNAs involved in cellular stress responses, inflammatory responses, and cell survival [85,86]. Therefore, the epigenetic alterations induced by Cd contribute to a range of health issues, including cancer, osteoporosis, and kidney disease, with Cd’s ability to silence tumor suppressor genes or activate oncogenes through epigenetic mechanisms notably facilitating the growth and dissemination of cancer cells [87]. Moreover, it may interfere with hormone signaling through epigenetic mechanisms, such as by altering the regulatory expression of genes related to sex hormones, impacting reproductive health (Figure 4) [88,89].

## 6. Conclusions and Future Perspectives

By summarizing the primary toxic effects and mechanisms of Cd, we can gain a deeper understanding of its extensive impact on human health. Cd poses a threat to health through various pathways, including disrupting intracellular calcium balance, inducing oxidative stress, disturbing cell signaling processes, and causing epigenetic effects. These toxic pathways are intertwined, and their cumulative effect makes it a highly destructive agent that leads to cellular dysfunction and tissue damage, particularly affecting vital organs such as the kidneys, bones, and liver.

Based on a comprehensive analysis of Cd toxic pathways and mechanisms, we propose the following recommendations: Given the persistence and bioaccumulation of Cd in the environment, stricter emission standards are essential to reduce environmental contamination and subsequent human exposure. This includes updating and tightening regulatory standards for Cd emissions in industrial processes, such as metal smelting and battery manufacturing. Regular monitoring can help identify contamination hotspots, assess the effectiveness of existing regulations, and provide data for timely intervention to protect public health. Therefore, implementing comprehensive monitoring programs to regularly assess Cd levels in air, water, soil, and food products, especially in areas near industrial activities, is crucial. Moreover, since Cd contamination is interconnected with other environmental issues like soil degradation and water pollution, integrating Cd management policies with broader environmental and public health policies ensures a holistic approach to pollution control and health protection. These strategies not only address immediate risks but also lay the groundwork for long-term prevention and sustainability.

Future research should continue to explore the specific toxic mechanisms of Cd to enhance our understanding of its impact on public health and to develop effective prevention and treatment strategies.

## Data Availability

Not applicable.
